# Supplementary material for: Transmon platform for quantum computing challenged by chaotic fluctuations
Source: Nat Commun. 2022 May 6;13:2495. doi: 10.1038/s41467-022-29940-y (PMC9076853; doi:10.1038/s41467-022-29940-y)
Supplement: Supplementary file 1 — Supplementary Information [file 41467_2022_29940_MOESM1_ESM.pdf]

Supplementary Information: Transmon platform for quantum computing challenged by chaotic fluctuations

Christoph Berke,[1] Evangelos Varvelis,[2,3] Simon Trebst,[1] Alexander Altland,[1] and David P. DiVincenzo[2,3,4]

[1]*Institute for Theoretical Physics, University of Cologne, 50937 Cologne, Germany*
[2]*Institute for Quantum Information, RWTH Aachen University, 52056 Aachen, Germany*
[3]*Jülich-Aachen Research Alliance (JARA), Fundamentals of Future Information Technologies, 52425 Jülich, Germany*
[4]*Peter Grünberg Institute, Theoretical Nanoelectronics, Forschungszentrum Jülich, 52425 Jülich, Germany*

SUPPLEMENTARY NOTE 1: EXPERIMENTAL FREQUENCY DISORDER

In the main manuscript we consider two principle approaches to the inclusion of frequency disorder in the design of transmon array devices, schemes A and B above. The fundamental difference between these two approaches is reflected in the dimensionless parameter $\delta\nu/t$, i.e. the strength of frequency disorder relative to the bare transmon coupling, which we have discussed as a proxy for the stability of many-body localization physics. Here we want to put the experimental approaches of Google and IBM into the context of these model classifications.

Let us start by summarizing typical parameters for IBM's cloud devices [1] which have been guiding us in the discussion of the main manuscript

- $\delta\nu = 70$–130 MHz
- $t \approx 3$ MHz
- $\delta\nu/t \approx 30$
- gate time: usually $\sim$ 400 ns, 100–200 ns are possible [2].

This design scheme is, at its core, geared towards *minimizing disorder* and has been dubbed scheme A in the main manuscript.

Before turning to its current generation of tunable coupler devices, Google's quantum devices [3] (as well as recent Delft chips [4]) operated at the order of

- $\delta\nu \approx 1$GHz
- $t = 30$ MHz
- $\delta\nu/t \approx 30$
- gate time: 40 ns

While tolerating a much stronger frequency spread (which brings this setting closer to design scheme B), the magnitude of the dimensionless parameter $\delta\nu/t \approx 30$ is close to what is seen in the IBM design above. An important distinction, however, is that the variability of the equally enhanced transmon coupling t is much more restricted, as it has to remain well below the absolute value of the typical charging energy $E_C = 250$ MHz. On the other hand, its already large value of $t = 30$ MHz leads to notably shorter gate operation times as in the IBM case.

These settings should be contrasted to the current generation of Google devices, such as the "Sycamore" processor (whose frequency disorder is visualized in Fig. 1). The introduction of tunable couplers [5] allowed Google to increase the relative strength of disorder by more than an order of magnitude. The device settings are [6, 7]

- $\delta\nu \approx 60$ MHz
- $t < 0.05$ MHz
- $\delta\nu/t > 1200$
- gate time: 12 ns

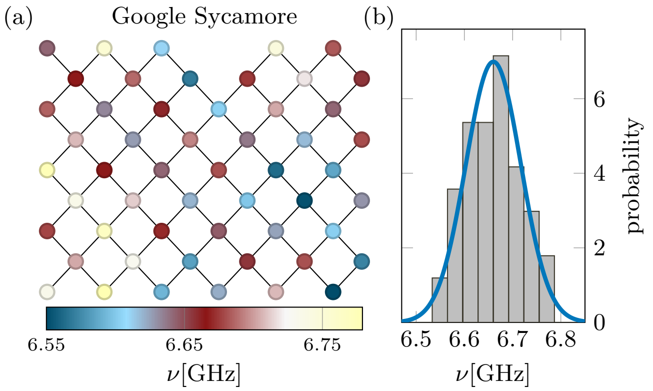

Supplementary Figure 1. **Experimental parameters of Google's transmon array.** (a) Layout of the 53-qubit transmon array "Sycamore". The coloring of the qubits indicates the variation of frequencies which is largely uncorrelated in space. (b) Spread of the frequencies plotted for the "Sycamore" chip, consistent with a Gaussian distribution (solid line).

This is a clear-cut example of design scheme B; a significant amount of disorder protecting the integrity of information via the principles of MBL.

SUPPLEMENTARY NOTE 2: SCHEME B DIAGNOSTICS

To complement our analysis for IBM's experimental parameters in the main text, let us consider parameters corresponding to scheme B, exemplified in recent Delft chips [4]. We summarize the three diagnostics of our analysis in Fig. 2. The phase diagrams of Fig. 2(a) show that the MBL-chaos transition has retracted to much larger T, with the KL calculation showing no significant departure from Poisson behavior. A minor drop in the IPR indicates dressing effects much smaller than in the scheme-A case. However, the Walsh diagnostic for a disorder realization with $\delta E_J = 7.5$ GHz, summarized in Fig. 2(b) and (c), shows that trouble is still around the corner: Higher-order terms of the τ-Hamiltonian are still present (albeit at a relatively lower level) and the ZZ danger threshold (dashed line) remains in sight. These values are observed for $T = 9$ MHz, about three times larger than for the natural-disorder parameters (scheme A) discussed in the main text. (Note that for the experimental transmon coupling of $t \approx 30$ MHz (corresponding to $T \approx 20$ MHz) the Walsh coefficient c_{11} (indicating the strength of the ZZ coupling) reaches a value in rather close agreement to the reported effective qubit-qubit coupling of $J_{\text{eff}} = 0.3$ MHz at the idle points in experiment [3].)

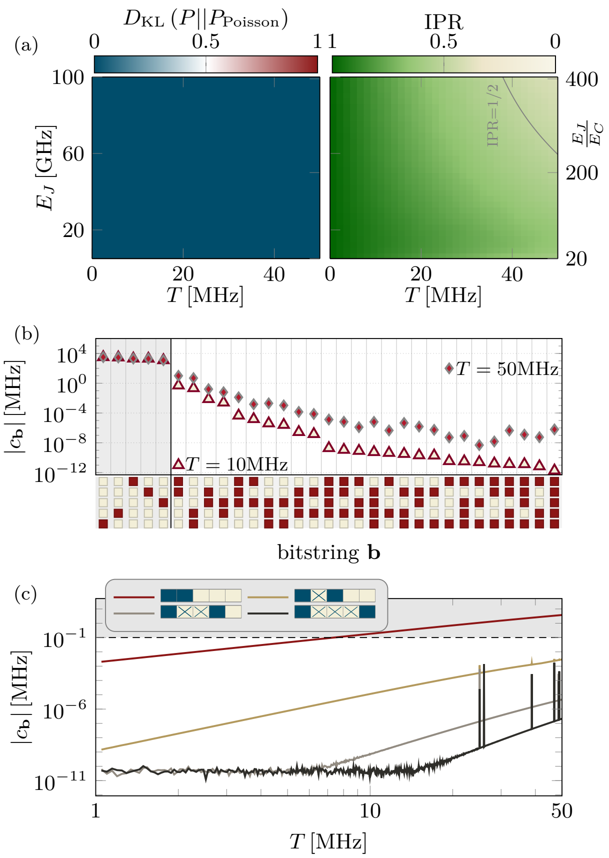

Supplementary Figure 2. **Summary of diagnostics for scheme-B parameters.** (a) Phase diagram in terms of spectral statistics (left) and IPR (right), akin to Fig. 4 in the main manuscript. (b) and (c) Walsh analysis, akin to Fig. 5 in the main manuscript. The results in (a) are averaged over at least 2000 disorder realizations. We use $E_C = 250$ MHz and $\delta\nu \sim 6E_C$, corresponding to $\delta E_J = \sqrt{18 E_C E_J}$. The spread of the Josephson energies thus varies from $\delta E_J \sim 3.4$ GHz for $E_J = 5$ GHz (E_J is bounded from below to ensure $E_J/E_C > 20$) to $\delta E_J \sim 15$ GHz for $E_J = 100$ GHz. The Walsh-transform analysis in (b) and (c) was performed for $E_J = 12.5$ GHz.

SUPPLEMENTARY NOTE 3: QUBIT FREQUENCY ENGINEERING

In its pursuit to engineer cleaner devices, IBM has recently introduced a two-step design [8] wherein the Josephson energy E_J (qubit frequency) is tuned by a laser-annealing technique "LASIQ" after the initial device fabrication process. The primary goal of this approach is to avoid frequency collisions [8, 9] (where, e.g., the transition frequencies of nearby qubits become degenerate).

In its current generation of cloud devices IBM employs LASIQ to enhance *anticorrelations*, $\Delta_{CT} = \nu_C - \nu_T$, where ν_C and ν_T are the qubit frequencies of nearest-neighbor transmons in the heavy-hexagon lattice geometry (denoted as "control" and "target" qubits). An overview of three devices of the current 27-qubit "Falcon", 65-qubit "Hummingbird" and 127-qubit "Eagle" generations is shown in Fig. 3. IBM has indeed succeeded in imprinting some of the desired anticorrelations (left column). However, the overall spread of qubit frequencies remains essentially Gaussian (right column). This being so, we expect that the results obtained in the main text hold including for these frequency engineered devices.

One may push the LASIQ technique to a next level by imprinting regular frequency *patterns* such as A-B or A-B-A-C into the heavy-hexagon lattice geometry currently used in all of IBM's cloud devices [1]. In this way, frequency crowding can be avoided [8, 10] (lowest panel of Fig. 3). Removing almost all random variations of the Josephson energy E_J, such layouts would implement the design philosophy A of the current manuscript in its purest form. As we will demonstrate below, a perfect realization of such a device would also remove the protective effects of many-body localization, and in this way compromise the integrity of quantum information.

SUPPLEMENTARY NOTE 4: PATTERN ENGINEERING.

To quantitatively discuss the role of MBL physics in the presence of engineered qubit frequencies, we consider an A-B sublattice pattern superimposed onto the 3×3 transmon lattice shown in Fig. 4(a). NN qubits are well separated in frequency, by an amount that has been deemed optimal for the operation of the cross-resonance scheme for performing entangling gate operations [12]. However, we note that precision engineering of alternating frequencies has a potentially problematic side effect: In a perfectly realized ... -A-B-A-... arrangement, weak but finite effective next nearest neighbor coupling between degenerate A (and B) transmons would lead to Bloch-band eigenstates. While this type of delocalization is not due to quantum chaos, chaos is in its wake the moment the inevitable presence of residual disorder is taken into account; in what amounts to a "fight fire with fire principle", the localizing counter effects of yet stronger disorder are required to stabilize the system.

To substantiate this picture, we apply our diagnostic tools to the 3×3 reference system, as summarized in Fig. 4. We identify four regimes (I-IV) distinguished by the value of random frequency detuning:

I. Global MBL phase for $\delta E_J > 0.1$ GHz.

II. Restructuring of the Hilbert space into energetically separated multiplets for 0.01 GHz $\lesssim \delta E_J \lesssim 0.1$ GHz.

III. Delocalization within these multiplet spaces for 10^{-4}GHz $\lesssim \delta E_J \lesssim 0.01$ GHz.

IV. Restructuring of the Hilbert space into 'molecular multiplets', reflecting the point group symmetries, for $\delta E_J < 10^{-4}$ GHz.

For very strong disorder, regime I, we have global MBL in a strongly coupled Fock space, as evidenced by the IPR approaching unity in Fig. 4(b), and the tangle of levels in Fig. 4(c).

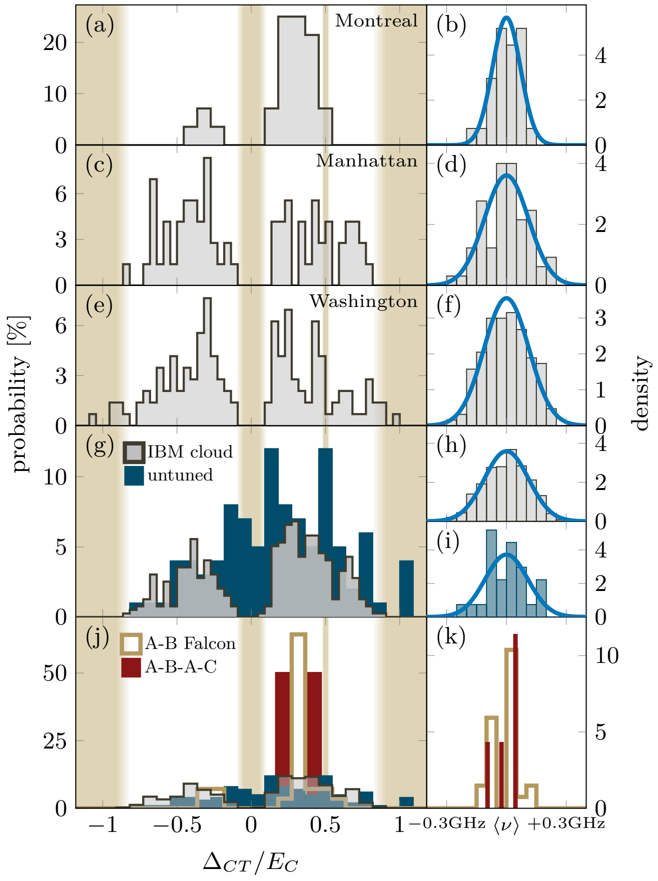

Supplementary Figure 3. **Overview of IBM cloud devices and LASIQ engineering.** Left column: distribution of NN frequency differences. Right column: distribution of frequencies around their mean values $\langle\nu\rangle$ (histograms) and fitted Gaussian distribution (solid line). Tanned areas mark the Δ_{CT} ranges where NN frequency collisions occur [8, 9]: at $\Delta_{CT}/E_C = -1, 0, 0.5, 1$. In (a)-(e) data for one chip of each of the three latest processor generations is shown [1]: Montreal ("Falcon"), Manhattan ("Hummingbird") and Washington ("Eagle"). The overall frequency spread is found to be consistent with a Gaussian, although each individual ν is specifically tuned to a desired value with high accuracy. In (g), the Δ_{CT} distribution for untuned transmons (blue) [11] is compared to the IBM cloud chips (gray, 9 Falcons, 2 Hummingbirds) [1]. There are significantly fewer collisions on the cloud devices compared to the untuned transmons, as a result of the LASIQ adjustments. (h) and (i) show the corresponding ν distributions which — despite the substantial differences in Δ_{CT} — are well described by Gaussians of similar width. In (j) and (k), data for 'pattern tuned' processors is shown, the 'A-B Falcon' from Ref. [11] that realizes an approximate A-B pattern (ochre), and the optimal 'A-B-A-C' pattern (red) [8]. These are the only configurations that show clear peaks in both the distribution for Δ_{CT} and ν. The bin width is 50 MHz for the right column. For the left column, the bin width is 1/22 for the IBM cloud chips in panel (c),(e),(g) and 1/11 otherwise.

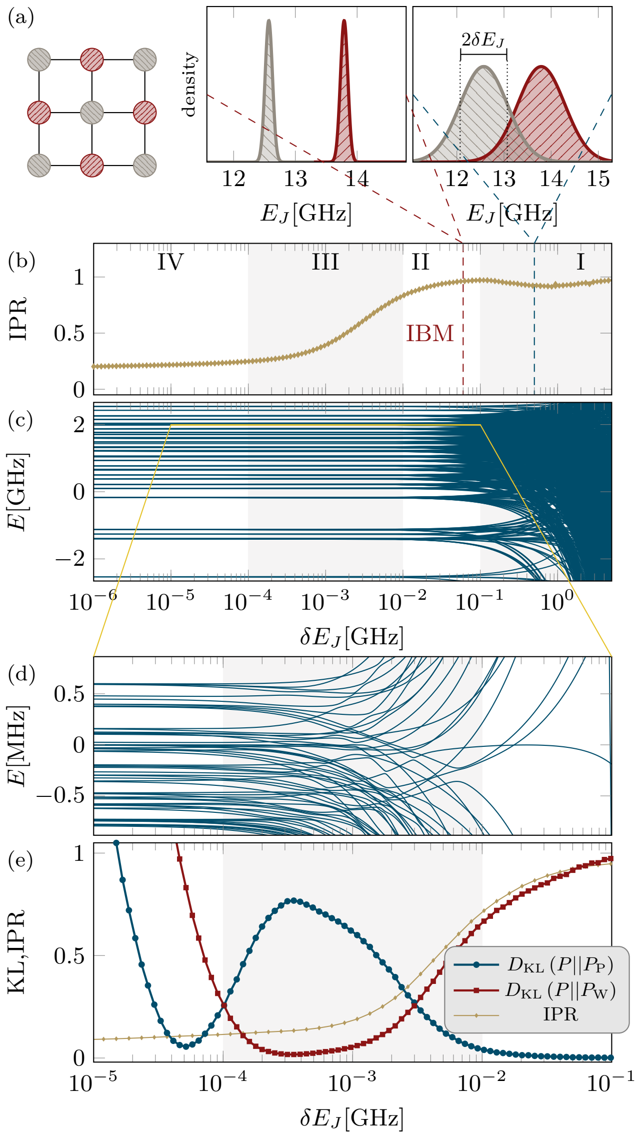

Supplementary Figure 4. **Effect of qubit frequency engineering.** (a) Staggered A-B sublattice arrangement of Josephson energies E_J in a 3×3 transmon layout subject to an adjustable disorder strength δE_J (right). (b) Inverse participation ratio versus δE_J. (c) Evolution of the 5-excitation bundle (with 1287 states) as a function of δE_J [13]. (d) Zooming into the single multiplet containing the 60 computational states defined by all permutations of three A transmons in $|1\rangle$ and two in $|0\rangle$, two B transmons in $|1\rangle$ and two in $|0\rangle$. (e) Spectral statistics of that multiplet quantified by the Kullback-Leibler divergence relative to Poisson and Wigner-Dyson distributions as in Fig. 3 in the main manuscript, and the inverse participation ratio (likewise computed for states inside the multiplet). The results in (b) and (e) are averaged over at least 8000 disorder realizations. Parameters were taken from Ref. [8]: on A sites (gray), we fix a mean Josephson energy $E_{J,A} = 12.58$ GHz, on B sites (red) $E_{J,B} = 13.80$ GHz. E_C is fixed to 0.33 GHz and $T = 3$ MHz, a typical low value of coupling favored in current experiments.

The energetically separated bundle structures that emerge in regime II, see panels (c) and (d), represent 'permutation multiplets', defined by a specific excitation structure on the two sublattices. The zoom in of Fig. 4(d) shows the levels

associated to a single multiplet (three A transmons in $|1\rangle$ and two in $|0\rangle$, two B transmons in $|1\rangle$ and two in $|0\rangle$, any permutation). There are 60 states in this particular permutation multiplet; note that they are all computational states. In this regime II – which is the one IBM operates in – states are efficiently localized *within* individual multiplets; the IPR is at a favorable value close to unity.

On the blown-up δE_J scale in (d), we see that levels are still strongly dispersing when the disorder is further reduced, but it is obvious by eye that we are now in a different situation, regime III, that is characterized by strong level repulsion. It is here that the IPR is dropping rapidly, meaning that the eigenstates are typically superpositions of states *within* this multiplet. This qualitative view is confirmed by our KL diagnostic in (e) showing that, in this region, the R_n distribution strongly resembles a Wigner-Dyson distribution – in short, we have entered a region of developed quantum chaos, a no-go area for quantum computation. Throughout this region, our Walsh-transform diagnostic is difficult to apply, because the assignment to definite computational states is ambiguous due to the strong multiplet mixing. This is the reason we do not show any Walsh-diagnostic results in this section.

As we proceed to even smaller disorder, there is yet a further evolution of the eigenspectrum: in regime IV the permutation multiplet resolves itself into a new set of bundles, which are now the molecular multiplets of the clean system. Here the remaining degeneracies are those of the point group symmetry of our 3×3 molecule. Reflecting the correlations introduced by these symmetries, the R_n distribution obeys neither Wigner-Dyson nor Poisson statistics in this regime.

Our analysis shows that the IBM work has currently landed in a good spot between too weak and too strong disorder. At this spot, the system is clean enough to preserve the identity of small sized (computational) subspaces, yet dirty enough to achieve state-localization within these spaces. We expect the insights obtained from the quantitative discussion above to be applicable to the more elaborated A-B-A-C scheme on the heavy-hexagon lattice: Despite the lower connectivity and the more complicated pattern, NNN qubits — the control qubits — remain degenerate.

SUPPLEMENTARY REFERENCES

[1] https://www.ibm.com/quantum-computing/ All calibration data was downloaded on 11/23/2021, except for the data for 'Washington' (12/15/2021) and the recently retired processors 'Manhattan' (11/16/2021) and 'Dublin' (11/08/2021).

[2] S. Sheldon, E. Magesan, J. M. Chow, and J. M. Gambetta, Procedure for systematically tuning up cross-talk in the cross-resonance gate, Phys. Rev. A **93**, 060302 (2016).

[3] R. Barends, J. Kelly, A. Megrant, A. Veitia, D. Sank, E. Jeffrey, T. C. White, J. Mutus, A. G. Fowler, B. Campbell, Y. Chen, Z. Chen, B. Chiaro, A. Dunsworth, C. Neill, P. O'Malley, P. Roushan, A. Vainsencher, J. Wenner, A. N. Korotkov, A. N. Cleland, and J. M. Martinis, Superconducting quantum circuits at the surface code threshold for fault tolerance, Nature **508**, 500 (2014).

[4] R. Versluis, S. Poletto, N. Khammassi, B. Tarasinski, N. Haider, D. J. Michalak, A. Bruno, K. Bertels, and L. DiCarlo, Scalable Quantum Circuit and Control for a Superconducting Surface Code, Phys. Rev. Applied **8**, 034021 (2017).

[5] F. Yan, P. Krantz, Y. Sung, M. Kjaergaard, D. L. Campbell, T. P. Orlando, S. Gustavsson, and W. D. Oliver, Tunable coupling scheme for implementing high-fidelity two-qubit gates, Phys. Rev. Applied **10**, 054062 (2018).

[6] F. Arute, K. Arya, R. Babbush, D. Bacon, J. C. Bardin, R. Barends, R. Biswas, S. Boixo, F. G. S. L. Brandao, D. A. Buell, B. Burkett, Y. Chen, Z. Chen, B. Chiaro, R. Collins, W. Courtney, A. Dunsworth, E. Farhi, B. Foxen, A. Fowler, C. Gidney, M. Giustina, R. Graff, K. Guerin, S. Habegger, M. P. Harrigan, M. J. Hartmann, A. Ho, M. Hoffmann, T. Huang, T. S. Humble, S. V. Isakov, E. Jeffrey, Z. Jiang, D. Kafri, K. Kechedzhi, J. Kelly, P. V. Klimov, S. Knysh, A. Korotkov, F. Kostritsa, D. Landhuis, M. Lindmark, E. Lucero, D. Lyakh, S. Mandrà, J. R. McClean, M. McEwen, A. Megrant, X. Mi, K. Michielsen, M. Mohseni, J. Mutus, O. Naaman, M. Neeley, C. Neill, M. Y. Niu, E. Ostby, A. Petukhov, J. C. Platt, C. Quintana, E. G. Rieffel, P. Roushan, N. C. Rubin, D. Sank, K. J. Satzinger, V. Smelyanskiy, K. J. Sung, M. D. Trevithick, A. Vainsencher, B. Villalonga, T. White, Z. J. Yao, P. Yeh, A. Zalcman, H. Neven, and J. M. Martinis, Quantum supremacy using a programmable superconducting processor, Nature **574**, 505 (2019).

[7] C. Neill, *A path towards quantum supremacy with superconducting qubits*, PhD Thesis, University of California (2017).

[8] J. B. Hertzberg, E. J. Zhang, S. Rosenblatt, E. Magesan, J. A. Smolin, J.-B. Yau, V. P. Adiga, M. Sandberg, M. Brink, J. M. Chow, and J. S. Orcutt, Laser-annealing Josephson junctions for yielding scaled-up superconducting quantum processors, npj Quantum Information **7**, 129 (2021).

[9] E. Magesan and J. M. Gambetta, Effective Hamiltonian models of the cross-resonance gate, Phys. Rev. A **101**, 052308 (2020).

[10] C. Chamberland, G. Zhu, T. J. Yoder, J. B. Hertzberg, and A. W. Cross, Topological and subsystem codes on low-degree graphs with flag qubits, Phys. Rev. X **10**, 011022 (2020).

[11] E. J. Zhang, S. Srinivasan, N. Sundaresan, D. F. Bogorin, Y. Martin, J. B. Hertzberg, J. Timmerwilke, E. J. Pritchett, J.-B. Yau, C. Wang, W. Landers, E. P. Lewandowski, A. Narasgond, S. Rosenblatt, G. A. Keefe, I. Lauer, M. B. Rothwell, D. T. McClure, O. E. Dial, J. S. Orcutt, M. Brink, and J. M. Chow, High-fidelity superconducting quantum processors via laser-annealing of transmon qubits (2020), arXiv:2012.08475 [quant-ph].

[12] J. M. Gambetta, Control of Superconducting Qubits, in, Proceedings of the 44th IFF Spring School, "Quantum Information Processing", Forschungszentrum Jülich (2013).

[13] Here is the procedure for creating the specific disorder realization for variable δE_J for our 3×3 array: Draw nine independent values v_i $(1 \leq i \leq 9)$ from the standard normal distribution $\mathcal{N}(0,1)$. Then the E_J value at site i is $E_{Ji} = 12.58\text{MHz} + v_i \delta E_J$ if site i is A type, and $E_{Ji} = 13.80\text{MHz} + v_i \delta E_J$ if site i is B type.
